# The emotional toll – personal and professional consequences of burnout in early career psychiatrists

**DOI:** 10.1192/j.eurpsy.2026.10266

**Published:** 2026-07-31

**Authors:** E. Chumakov

**Affiliations:** Department of Psychiatry and Addiction, Saint-Petersburg State University, Saint-Petersburg, Russian Federation

## Abstract

**Abstract:**

Burnout among early career psychiatrists represents a critical and often overlooked crisis that threatens the future of mental healthcare delivery. This presentation examines personal and professional consequences of occupational stress in early career psychiatrists who navigate the challenging transition from training to independent practice. The paradox of our profession is that those entrusted with safeguarding mental health frequently neglect their own psychological well-being, with early career practitioners being particularly vulnerable due to idealistic expectations, inadequate preparation for emotional demands, and the pervasive stigma surrounding help-seeking within medical culture. Personally, burnout manifests through emotional exhaustion, depersonalization toward patients, and a diminished sense of personal accomplishment, with alarming rates of anxiety, depression, and suicidal ideation reported among this population. The shame and fear of professional repercussions often prevent early career psychiatrists from seeking the very support they provide to others, creating a dangerous cycle of silent suffering. Professionally, the consequences extend beyond individual practitioners to directly compromise patient care: burnout correlates with reduced empathy, increased diagnostic errors, poorer therapeutic alliances, and higher rates of suboptimal treatment decisions. At the systemic level, burnout drives premature attrition from the field, contributing to workforce shortages precisely when demand for mental health services is escalating globally. This presentation argues for urgent, multi-level interventions including mandatory supervision and mentorship programs, institutional normalization of help-seeking behaviors, curriculum reforms addressing emotional preparedness, and the destigmatization of vulnerability within psychiatric training. Protecting the psychological health of early career psychiatrists is not merely an occupational health issue but a fundamental patient safety imperative. We cannot effectively heal others when we ourselves are wounded and silenced.

**Disclosure of Interest:**

None Declared

